# Mammographic screening debate on study design: a need to move the field forward

**DOI:** 10.1186/1741-7015-10-164

**Published:** 2012-12-12

**Authors:** Giske Ursin

**Affiliations:** 1 Cancer Registry of Norway, Majorstuen, N-0304 Oslo, Norway; 2 Institute of Basic Medical Sciences, University of Oslo, Blindern, 0317, Oslo, Norway; 3 Department of Preventive Medicine, University of Southern California, Los Angeles, CA, USA

**Keywords:** breast cancer, mammographic screening, study design

## Abstract

The mammographic screening debate has been running for decades. The temperature of this debate is unusually high, and all participants, regardless of viewpoint, seem to have a conflict of interest. Another unusual aspect of this debate is the focus on study design, and in particular on designs that some think exceeded their usefulness decades ago. What are the questions that remain to be answered in this debate? Are there methodological issues that have not been adequately addressed? Do we have the right tools to provide up-to-date answers to how women can best protect themselves against dying from breast cancer? This commentary discusses some of the current issues.

See related Opinion articles http://www.biomedcentral.com/1741-7015/10/106 and http://www.biomedcentral.com/1741-7015/10/163

## Background

Over the past decades, much has been written about the benefits and harms of mammographic screening. Although many debates have raged in medicine, this debate has certain bizarre aspects. For one, the temperature has always been exceedingly high. It has been argued that this is because the proponents have their livelihood dependent on screening, and thus strong conflicts of interest. Their opponents, on the other hand, might claim they are simply defending women, and specifically, women's breasts. Or are they? Careers have been built around this sole issue: defending the integrity of women's breasts. Perhaps we will not move forward until we recognize that both sides have conflicts of interest. As such this area is no different than many other areas of medicine.

Consistent with this was the definition of 'independent' of the recently converged independent panel in the UK that were asked to evaluate the benefits and harms of mammographic screening. In their report [1], the authors explained their independence by the fact that none of them had previously published on mammographic screening. Thus, apparently, only virgin writers are truly independent in this debate.

Another strange aspect of the screening debate is the discussion of study design, exemplified by two recent papers in *BMC Medicine *[2,3]. Based on what we teach our medical and public health students, this debate makes little sense. Our students are taught that there is a strong hierarchy in study designs. Randomized clinical trials are time consuming and expensive, but represent the gold standard. Thereafter come properly performed cohort studies, then modern (incidence-based) case-control studies, then the cumulative-based case-control studies and cross-sectional studies. At the bottom of the list are the ecologic studies, studies based on aggregate numbers on a population, such as studies of trends over time. These studies have well described biases. Conclusions drawn from these studies should not, according to textbooks in epidemiologic methods [4], be used to draw inferences at the biological or individual level.

Researchers in medicine and public health tend to move up in this hierarchy of study designs when addressing a hypothesis, although not all hypotheses reach clinical trials. In mammographic screening, the trials were performed more than 30 years ago. Because these found that screening reduced mortality of breast cancer, several countries introduced organized screening programs [5]. The question then became whether we would see as strong protective effects in the screening programs in the general population as had been reported from the clinical trials. The women who participate in clinical trials may differ from the population at large on a number of key prognostic variables, partly because of the strict exclusion criteria in clinical trials. The normal procedure in medicine is then to conduct observational studies, such as cohort or case-control studies with individual data to address this issue.

However, one of the ongoing debates in mammographic screening is puzzling, and it is difficult to understand why this would move the field forward. The discussion is not whether observational studies confirm the effect found in clinical trials, in fact there seems to be agreement that they do. Instead it is the bottom part of the hierarchy of study designs that is being discussed. There are actual proponents for the bottom level design, as exemplified by Autier and Boniol [3]. Their argument is simply put that because cohort studies and case-control studies can be biased, we should use ecological studies. The problem with this argument is that if cohort and case-control studies can be biased, ecological studies have worse problems, as they do not have information on each individual [4]. Individuals are not classified correctly and even strong associations can be missed. The UK panel [1] simply ignored these studies in their summary as being 'not helpful'.

Puliti and Zappa [2] argue that studies with data at the individual-level (case-control and incidence-based mortality studies) should be used. This is a conclusion that fits with the classical hierarchy of study designs, and therefore may seem obvious. However, even so it is important to acknowledge the limitations of both case-control and cohort studies and to better understand how the biases work in such study designs, and how they should be corrected.

Autier and Boniol [3] discuss one important bias, the self-selection bias in case-control and cohort studies. Screening participants are healthier than non-participants. Thus even in the absence of screening, the screening participants would have had lower mortality than non-screening participants. The issue is how to correct for this. Autier and Boniol show an example of when the correction proposed by Duffy and colleagues [6] can go wrong. This is a good start, but Autier and Boniol did not include the most obvious solution to this problem. Those who conduct case-control or cohort studies simply need to be sensible about their adjustment. If the estimated correction factor from one's study is unreasonably low compared to previous estimates, then be cautious, as it may be wrong, and one needs to discuss how the results change if the correction factor changes. In Autier and Boniol's paper, the correction factor in their example (D_r _in their paper, relative risk of breast cancer death for non-compliers compared to a non-invited comparison group) of 1.07 was too low, but if the investigators had used Duffy *et al*.'s estimate based on clinical trials, which was 1.36 [6], the bias would have been minimal.

The recently published UK report [1] concluded that although most case-control studies and cohort studies tried to control for possible biases, and although the results were in the same direction as the randomized clinical trials, there was some concern that residual bias could have inflated the estimates from these studies. In the end the UK panel only used results of randomized clinical trials, the most stringent in the hierarchy of study designs.

The independent UK panel concluded that 'screening reduces breast cancer mortality but that some overdiagnosis occurs' [1]. Specifically they concluded that screening confers a 20% reduction in breast cancer mortality, and that the overdiagnosis is 19% during the screening period or 11% over long term. The main challenge is that while all detected cancers will be treated, we do not yet know which of the cancers would kill the woman if left untreated. Although we have learned a substantial amount about breast cancer subtypes and prognosis over the past 12 years, the number of subtypes continues to rise [7]. We do not yet have adequate clinical markers for these subtypes, or even prognostic markers that can accurately classify a patient as needing treatment or not. There is a strong need for additional screening methods to better differentiate cancers and specifically identify the cancers that will kill.

In assessing the overall effect of screening on mortality, the independent UK panel concluded that the best studies were old trials, studies conducted 30 or so years ago. Much has changed in both breast cancer risk factors and treatment over time. Are data from when the current screening participants were in their teens ideal to express what these women should do to avoid dying from breast cancer today? How long should we continue to use such old data as the best estimate for how mammographic screening affects breast cancer mortality? When will both sides agree that this is unwise?

The obvious alternative to rehashing old data is to force screening proponents and opponents to work together to identify the best way of using current individual based data to address the issues. A challenge is whether we have identified all the limitations of our best observational analytic designs, that is, cohort and case-control studies, and when current bias adjustments are inadequate. If the pro-screening side ignores these biases, or both sides present them too simplistically, then we will get nowhere. We ought to instead create scenarios that both sides recognize represent real problems with cohort and case-control studies, and then address each specific problem analytically, rather than going back to ecological designs.

## Future directions and conclusions

Currently there are a number of new diagnostic methods being introduced in the clinic, and an array of possible prognostic or subtype markers that may reach the clinic soon. Although, this has the potential of improving breast cancer screening, we will need to evaluate which combination of screening and prognostic markers are cost efficient in large screening programs. Such evaluations can be performed in stringent trials, but there is a strong need for us to do this continuously in observational studies based on individual-level data. It will then be important that we have the adequate epidemiological and statistical tools to test these stringently. To move forward in this endeavor, it is useful with a continuous debate of these issues in mammographic screening, as long as the debate focuses on the real issues, and not on whether one's side is right or wrong.

## Competing interests

GU has since 2011 been the director of the Norwegian Cancer Registry, which is responsible for the Norwegian Breast Cancer Screening Program.

## Pre-publication history

The pre-publication history for this paper can be accessed here:

http://www.biomedcentral.com/1741-7015/10/164/prepub
